# Proteomic Investigations of Two Pakistani *Naja* Snake Venoms Species Unravel the Venom Complexity, Posttranslational Modifications, and Presence of Extracellular Vesicles

**DOI:** 10.3390/toxins12110669

**Published:** 2020-10-22

**Authors:** Aisha Manuwar, Benjamin Dreyer, Andreas Böhmert, Anwar Ullah, Zia Mughal, Ahmed Akrem, Syed Abid Ali, Hartmut Schlüter, Christian Betzel

**Affiliations:** 1 Department of Chemistry, University of Engineering and Technology, Lahore 54890, Pakistan; 2 Institute of Clinical Chemistry and Laboratory Medicine, Mass Spectrometric Proteomics, University Medical Centre Hamburg-Eppendorf (UKE), Martinistraße 52, 20246 Hamburg, Germany; b.dreyer@uke.de (B.D.); andy.boehmert@googlemail.com (A.B.); hschluet@uke.de (H.S.); 3 Department of Biosciences, COMSATS University Islamabad, Park Road, Chack Shahzad, Islamabad 45550, Pakistan; anwar.ms90@yahoo.com; 4 Pet Centre, University of Veterinary and Animal Sciences, Lahore 54890, Pakistan; zia_mgl@hotmail.com; 5 Botany Division, Institute of Pure and Applied Biology, Bahauddin Zakariya University, Multan 60800, Pakistan; ahmedakrem@bzu.edu.pk; 6 Husein Ebrahim Jamal Research Institute of Chemistry, (International Center for Chemical and Biological Sciences), University of Karachi, Karachi 75270, Pakistan; abid.ali@iccs.edu; 7 Laboratory for Structural Biology of Infection and Inflammation, Institute of Biochemistry and Molecular Biology, Deutsches Elektronen-Synchrotron, Build. 22a, Notkestr. 85, University of Hamburg, 22603 Hamburg, Germany

**Keywords:** *Naja naja*, *Naja oxiana*, venom proteome, Ras-GTPase, ankyrin repeat, N-terminal acetylation, extracellular vesicles

## Abstract

Latest advancement of omics technologies allows in-depth characterization of venom compositions. In the present work we present a proteomic study of two snake venoms of the genus *Naja* i.e., *Naja naja* (black cobra) and *Naja oxiana* (brown cobra) of Pakistani origin. The present study has shown that these snake venoms consist of a highly diversified proteome. Furthermore, the data also revealed variation among closely related species. High throughput mass spectrometric analysis of the venom proteome allowed to identify for the *N. naja* venom 34 protein families and for the *N. oxiana* 24 protein families. The comparative evaluation of the two venoms showed that *N. naja* consists of a more complex venom proteome than *N. oxiana* venom. Analysis also showed N-terminal acetylation (N-ace) of a few proteins in both venoms. To the best of our knowledge, this is the first study revealing this posttranslational modification in snake venom. N-ace can shed light on the mechanism of regulation of venom proteins inside the venom gland. Furthermore, our data showed the presence of other body proteins, e.g., ankyrin repeats, leucine repeats, zinc finger, cobra serum albumin, transferrin, insulin, deoxyribonuclease-2-alpha, and other regulatory proteins in these venoms. Interestingly, our data identified Ras-GTpase type of proteins, which indicate the presence of extracellular vesicles in the venom. The data can support the production of distinct and specific anti-venoms and also allow a better understanding of the envenomation and mechanism of distribution of toxins. Data are available via ProteomeXchange with identifier PXD018726.

## 1. Introduction

**Figure 1:**
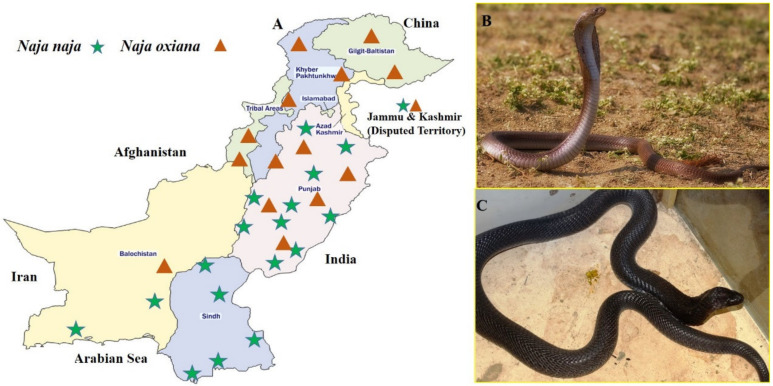
(**A**) Geographical distribution of the genus *Naja* snakes in Pakistan. (**B**) *Naja oxiana* (Brown cobra) (**C**) *Naja naja* (black cobra).

Pakistan has a particular geographical location and hosts an array of habitats such a, mountains, glaciers, coastal areas, swamps, plane areas, fresh water, and sandy areas [1]. The country is located between two zoogeographical regions (Palearctic and Oriental) and hosts a diverse venomous fauna. Nine habitat zones are recognized according to the distribution of snakes in Pakistan [2]. Seventy-two snake species are known to Pakistan, among which 14 marine and 12 terrestrial are venomous [1]. According to ITIS (Integrated Taxonomic Information System) database there are 29 snake species belonging to the genus *Naja* [3]. Among these two are found in Pakistan, i.e., *Naja naja* and *Naja oxiana* [2]. Both of these snakes are non-spitting cobras [4]. These snakes are shy of humans. However, upon assessing threat they lift the anterior part of their body, display a hood, and if provoked, hiss loudly and sway their hood to frighten their adversary. These snakes attack very furiously, chewing the bitten part. They usually feed on rodents, birds, frogs, lizards, and snakes. They are found in rocky, stony foothills, forests and around the villages [2]. *N. naja* (black cobra) is known to have variable color and pattern. However, in Pakistan juveniles and young adults tend to be grey with hood marks, but the adult specimens are usually uniformly black. In addition, the throat pattern is obscured in adult snakes, due to pigmentation [4,5]. *N. naja* is distributed in North West Pakistan, south and desert areas, except most of Baluchistan. *N. oxiana* (brown cobra) occurs sympatrically in the Northern half of Pakistan with *N. naja*. Adult *N. oxiana* is normally brown in color [5,6]. These snakes and their geographical distribution are shown in Figure 1.

Only a few reliable data exist reporting the frequency of morbidity because of snakebites in developing countries. However, it is predicted that snakebite is responsible for a substantial amount of morbidity and mortality in remote areas [7]. The hidden toll of suffering continues to affect the families of the deceased, and patients who survived with crippling deformity [8]. World Health Organization (WHO), included snakebite in its list of “Neglected Tropical disease” in 2007 [9]. Recently, WHO also added snakebite envenoming at high preference in the list of Neglected Tropical disease, in 2017, upon request of some member states of United Nations. The supply of antivenom and snakebite management was declared as a global public health emergency. WHO has included snake antivenom immunoglobulins in the “WHO Model List for Essential Medicines” WHO has also encouraged countries to ensure their national antivenom stocks [10] Despite these efforts, snakebite has not gained attention on international public health agendas [11]. The snakes commonly responsible for clinically significant bites in Pakistan are *Bungarus caeruleus* (common krait), *N. naja* (cobra), *Daboia russelii* (Russel’s viper), and *Echis carinatus* (Saw-scaled viper) [12]. National Institute of Health, Pakistan, produces around 30,000 vials of polyvalent anti-venom per year. However, the amount of this antivenom is not sufficient and can only treat a fraction of snakebite cases in the country (https://www.nih.org.pk/1255-2/) [13]. To meet the requirement of antivenoms, snake antivenom sera are presently also imported from India. However, studies have shown that Indian antivenoms provide partial neutralization, particularly for *N. naja* venom [14,15,16]. Although, *N. naja* and *N. oxiana* are also prevalent in India, but the venom composition is known to vary within the same species, due to change in geographical and ecological factors [17,18,19,20]. A study reported that Pakistani *N. naja* is more neurotoxic with lower LD_50_, then that prevalent in India [8]. Gender, diet, and age of the snake is also known to influence the composition of venoms [21,22,23,24].

Depending on the amount of venom injected, paralysis following cobra bites can occur within several hours, with death ensuing if breathing is not assisted [8]. On average cobras can inject 60 mg of venom in a bite [25]. Cobra venom is a postsynaptic neurotoxin and presents a variety of symptoms like pain, edema, necrosis, respiratory paralysis, headache, cardiac arrest, hypotension, and bleeding wounds [26]. The use of anticholinesterase, such as neostigmine, has been suggested to compensate a cobra bite, in addition to the administration of antivenom [25,26].

Recent scientific advances have paved the way to explore venomous snake composition in detail and various strategies have been evolved to better understand venom components, their function and immunological properties [27]. Genomic and transcriptomic studies have proved to be an invaluable tool in the discovery of the snake venom evolution and proteoform [28,29,30,31,32,33,34]. Consequently, investigations are directed towards the discovery of pharmacologically active snake venom compounds [35,36,37,38]. For example, a recent study reported Mambaquaretin-1 (peptide from green mamba venom), as a promising candidate for the treatment of polycystic kidney disease [39]. Another study described Nubein6.8, a peptide from the venom of *N. nubiae*, as a promising template for the treatment of human melanoma and ovarian cancer [40].

In the present study, we describe an in-depth comparative proteomic study of two Pakistani snake species of the family elapid and genus *Naja*, i.e., *N. oxiana* (brown cobra/Caspian cobra/Central Asian cobra) and *N. naja* (black cobra/Indian cobra/Spectacled cobra). In Pakistani region both species of adult cobras are melatonic and *N. oxiana* is commonly known as brown, while *N. naja* is known as black cobra. These snakes were previously known as *Naja n. oxiana* and *Naja n. karachienis* respectively, but now they are named according to the ITIS database [41]. Till now only a few studies have been reported about the proteomics of Pakistani *N. naja* [42,43,44]. The *N. naja* venom samples in these studies were collected from Southern Punjab and Sindh Province of Pakistan. These research groups performed pre fractionation of the venom sample either by reverse phase chromatography, 1-dimensional gel electrophoresis (1D gel) or 2-dimensional gel electrophoresis (2 D gel) or a combination of these methods. Further mass spectrometric analysis of peptide fragments obtained from in gel trypsin digestion, was carried out by MALDI TOF/TOF, ion trap or ESI MS. Chanda et al. also reported the venom proteomics of *N. naja*, from Western and Eastern parts of India [45,46]. In their study of the venom sample from East India, they pre fractionated the crude venom by 1D gel prior to LTQ orbitrap analysis. However, the proteomic analysis of the venom sample from Western India was performed by a combination of fractionation methods and LC- MS/MS was done by QTOF mass spectrometer. Analysis of the comparative statement of the research group showed that pre fractionation of the crude venom by gel filtration chromatography followed by gel electrophoresis, worked best in their hands. The same group reported the proteomic study of South Indian *N. naja* venom, recently [47].

In this work, they separated the crude venom components by 1D gel electrophoresis. The mass spectrometric analysis of the tryptic peptide was performed on QTOF. The results of this study derive a comparison of common and unique toxins in *N. naja* venom obtained from all the three different Indian regions. Our results revealed remarkable differences in the relative abundance of the venom components, as compared to the previous studies. In addition, our investigations unveiled new venom components, not reported before in these venoms. The variation in the results could be different geographical of the snakes from which we collected the venom samples. Further, our workflow did not involve any pre fractionation of the venom. Pre fractionation by gel electrophoresis or liquid chromatography might lead to the loss of some low abundant venom components. Also, we used a modern version of the orbitrap mass spectrometer in this work which is very sensitive equipment.

To the best of our knowledge, this is the first report on the proteomic study of *Naja oxiana* venom. The abbreviations used for proteins and peptides are given in Table 1.

## 2. Results

**Figure 2:**
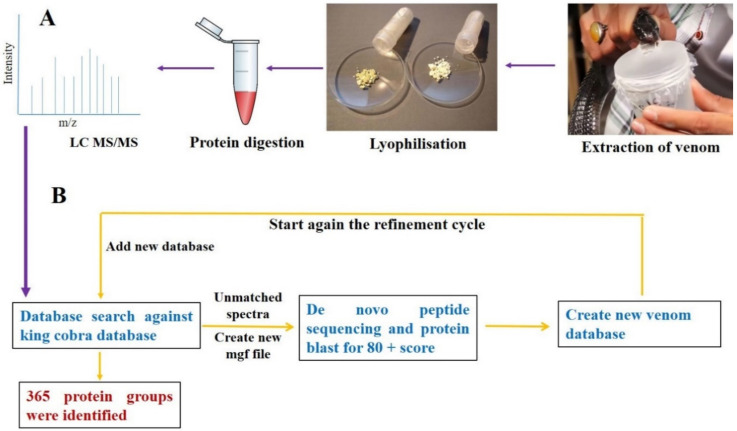
(**A**) Milking of *N. naja* venom and sample preparation for LC-MS/MS analysis (**B**) Data base search cycle.

The venom proteome of *N. naja* (NN) and *N. oxiana* (NO) snakes was investigated by mass spectrometric analysis, using a shotgun proteomic approach. We were able to provide an extensive overview of various protein families present in both venoms, based on data base searches and BLAST analysis of the de novo sequenced tandem mass spectra. A total of 735 peptides from NN and 254 peptides from NO were sequenced (Supplementary Table S1 and S2). Subsequently 365 proteins in NN venom (Table 2) and 140 proteins were identified in NO venom (Table 3). The sequences of the protein fragments are listed in Supplementary Tables S1 and S2. The results obtained allowed us to cluster the venom protein content into 34 protein families for *N. naja* and in 24 protein families for the *N. oxiana* venom. Figure 2A illustrates the preparation for MS acquisition and Figure 2B represents the strategy applied for data base searches. In the present work, we performed data base search against Serpents, King cobra utilizing Uniprot data base. The venom of *Ophiophagus hannah* has been well studied and genomic and proteomic data are available in the database [28,48,49]. A recent study showed similarity between the genome of Indian cobra and King cobra [50]. This group analyzed 139 *N. naja* venom gland toxin genes to identify orthologs in the King cobra. It was determined that 96 genes matched while 43 did not. It was suggested that, although some genes are likely to be unique to Indian cobra, the majority were not annotated in King cobra genome. The possible reason could be its highly fragment assembly. Based on this similarity, we searched our data against King cobra database also. Further, in the data base complete proteome of only King cobra is available. The details of our search against Serpent database are presented in Supplementary Table S3 and S5 while that against King cobra are compiled in Supplementary Table S4 and S6. The results presented and discussed are a conclusion of both data base searches.

**Figure 3:**
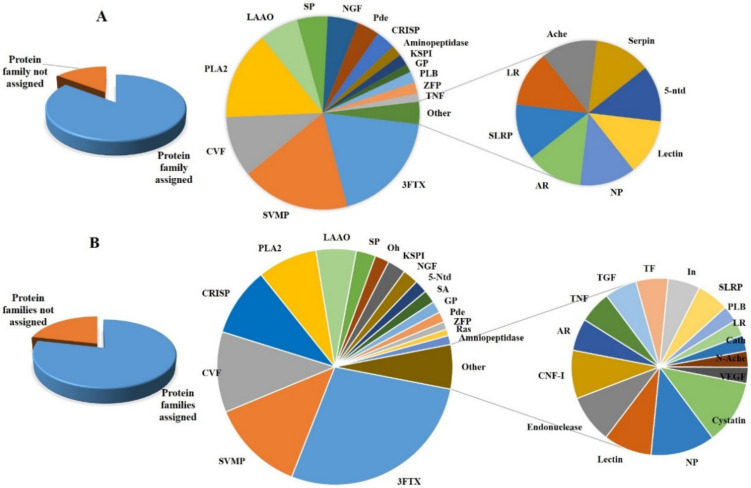
Pie chart illustrations highlighting the relative abundance of various protein families in the two venoms. (**A**) *N. oxiana* (**B**) *N. naja.*

A comparative summary of the protein families of the two venoms is presented in Table 1. Figure 3, presents a comparison of the relative abundance of different venom protein families as pie charts. From the pie charts, it can be observed that there are significant differences in the proteome of two snake venoms. In the venom of *N. naja*, three-finger toxins (3FTx) are more abundant, while in *N. oxiana* venom, both 3FTXs and snake venom metalloproteinase (SVMPs) are almost equally abundant. In NO, snake venom serine proteases (SVSPs) and phospholipase A_2_ (PLA_2_s) are much more abundant than in NN. There are other subtle variations in the relative abundance of protein families between the two venoms. For example, Cysteine-rich Secretory Protein (CRISP) family is much more abundant in NN as compared to NO. Further, NN venom contains 11 protein families, which could not be found in NO venom, listed in Table 1 and highlighted in red color. Whereas NO venom contains serpins, which are absent in NN venom. Figure 3 shows that NN venom is much more versatile and contains a number of different proteins (Table 1). Data base searches revealed that our data provide a deeper insight of the NN and NO venom proteomes. There are several protein families, which have not been reported earlier in NN venom, including western and eastern Indian *N. naja*. In Table 1, the protein families discovered and reported for the first time in terms of our investigations are shown with check mark (✓). Interestingly previous studies reported PLA_2_ as the second most abundant protein family found in *N. naja* venom, and that SVMP was present in relatively low abundance [8,42,43,44,45,46]. In contrast, our data showed SVMP as the second most abundant protein family in *N. naja.* The venom proteome of *N. oxiana* displays that, both 3FTXs and SVMP are equally abundant like that of king cobra *(Ophiophagus hannah)* [51], as illustrated in Figure 3A.

In the present work, three types of posttranslational modification were also observed, i.e., N-terminus pyro-glutamate, methionine oxidation and N-terminal acetylation (N-ace). Pyro-glutamate posttranslational modifications of the venom proteins has been described before and are known to confer stability to the proteins and peptides [52,53,54,55]. However, modification of methionine residues and pyro-glutamate cannot be excluded during sample preparation. Therefore, keeping in view this possibility we have not discussed the observed methionine and pyro-glutamate modifications. The current study is the first description of N-terminal acetylation of venom proteins. In *N. naja* venom we were able to identify three peptide fragments (Muscarinic toxin-like protein 3, Acid phospholipase A_2_ and weak neurotoxin 7) containing N-ace modification. Whereas in *N. oxiana* one peptide (Muscarinic toxin-like protein 3) was identified with N-ace. These sequences have been highlighted with green colour in Supplementary Tables S1 and S2.

In the present work, we have identified a number of proteins like cobra serum albumin, leucine repeat, zinc finger containing protein, venom lectin protein, Ras-like protein. The presence of Ras-like protein demonstrates the presence of extracellular vesicles in the venom of *Naja naja.* The comparison of our proteomic data with that of *N. naja* snake both from western and eastern India, reveals that such proteins were not identified in Indian *N. naja*, Further in Pakistani *Naja naja* snake we not could identify cholinesterase, butyrylcholinesterase, hyalurinidase and snaclec proteins which were previously reported in Indian *N. naja* venom [45,46].

Below we briefly describe and discuss the different venom protein families identified.

## 3. Discussion

### 3.1. Major Protein Components (Relative Abundance >2%)

#### 3.1.1. Three-Finger Toxins

**Figure 4:**
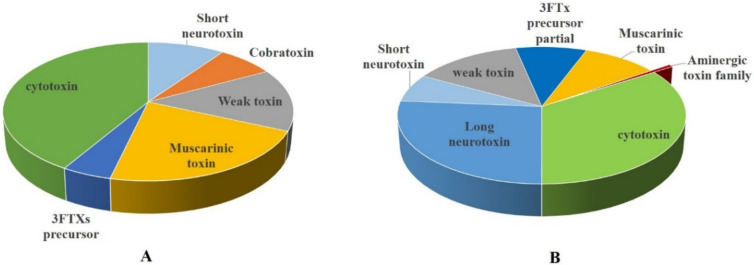
Pie chart illustrating a comparative profile of the three-finger toxins present in the two snake venoms. (**A**) *N. oxiana* (**B**) *N. naja*.

The detailed proteomic investigations of the, NN and NO snake venoms identified two main types of three-finger toxins, i.e., neurotoxins and cytotoxins. The venom of NN consists of an overall higher abundance and a greater diversity of 3FTXs, as compared to NO (Table 1 and Table 2, Figure 3). Neurotoxins are predominant in both venoms, as compared to cytotoxins, Figure 4. Among the neurotoxins, long, muscarinic, weak, 3FTxs precursor and aminergic toxin families are present in both venoms. In case of NO, a rather low amount of long neurotoxin is present, represented by one neurotoxin, cobratoxin. Whereas, long neurotoxins constitute a major proportion of neurotoxins found in the NN venom. Figure 4 indicates that in NO venom, muscarinic toxins are present in relatively higher amounts as compared to NN venom. It is interesting to note that NN venom contains an aminergic neurotoxin with homology to *Dendroaspis angusticeps* venom toxin AdTx1. This toxin is known to impair G-protein-coupled receptors [56,57].

Previous studies have shown that 3FTXs make up approximately 56–84% of venom proteome in various species of *Naja* [58]. However, our results of Pakistani *Naja* venom samples show a much lower percentage of 3FTXS as compared to other investigations, which is 21% in NN and 16% in NO of the total venom proteins. In contrast to Pakistani *N. naja* venom proteome, the eastern Indian *N. naja* venom comprises of 61% 3FTXs and western Indian *N. naja* contains 68% 3FTXs. Interestingly eastern Indian *N. naja* lacks short neurotoxins, which are present in both western Indian and Pakistani *N. naja* snake venom [45,46].

Investigating 3FTXs are not only of interest to characterize their toxicity, but also of great significance for structural studies, as well as for biotechnological, biomedical and evolutionary studies [59,60,61,62,63]. Already, 3FTXs have proven to be an efficient tool to analyze various receptor types, and to study diseases like Parkinson’s disease, myasthenia gravis and cancer [64,65,66,67,68,69,70,71]. The aminergic toxins from mamba venom served as good candidates for protein resurrection methodology [72].

#### 3.1.2. Phospholipase A_2_

Both *Naja* snake venom contain PLA_2_. The percentage abundance of PLA_2_ enzymes (12.6%) is higher in NO as compared to NN. PLA_2_s make up 6% of the venom of NN (Table 1). A recent study reported the comparative enzymatic activity of PLA_2_s in ten different *Naja* species, with highest activity in *N. siamensis* and lowest in *N. nivea* [73]. The venom proteome study of Indian *N. naja* venom carried out by A. K. Mukherjee research group reported that Indian *N. naja* contains 20–27% PLA_2_s [45,46]. This is much higher than the amount of PLA2s present in Pakistani *N. naja*. A proteomic study of *N. kaouthia* venom reported PLA_2_s as one of the most abundant venom proteins [74]. While another study on the venom proteome of *N. annulifera* did not detect PLA_2_s [75]. In the venom of *N. philippinensis* PLA_2_s made up 22.88% of venom proteome [76]. Another study showed distinct distribution of PLA2s in Afro-Asian cobra venom. Asian spitting cobras showed highest PLA_2_ activity. Asian non-spitting and African spitting cobras showed moderate activity and low activity was shown by African non-spiting cobras [77].

Table 3 shows that both venom comprise of acidic and basic PLA_2_s. However, acidic PLA_2_s are more abundant in the two venoms. Two fragments of phospholipases from NO bear homology to neutral PLA_2_s paradoxin-like beta chain from *Oxyuranus microlepidotus*. This protein was found to be one of the most potent presynaptic neurotoxins [78]. Eleven peptide fragments bearing homology to acidic phospholipase in the venom of *Naja sputatrix* were identified (Table 3). In the *Naja naja* peptide fragments having homology to acidic PLA_2_s from the venom of other *Naja* species were determined (Table 2). Six peptide fragments showed homology to acidic PLA_2_ from the venom of *Pseudonaja textilis*. A previous study reported this molecule to have moderate enzymatic activity and procoagulant property and was found to be non-lethal [79]. In the NN venom two peptide fragments matching Basic phospholipase A2 from *Bungarus candidus* venom and one matching with basic PLA_2_ with sea krait was identified. While in NO venom only one peptide fragment having homology to a basic PLA_2_ from *Bungarus candidus* was found. The activity and specificity of basic phospholipases from *Agkistrodon h. blomhiffii* and Pakistani *N. naja* was studied on intact human erythrocytes. Although belonging to two different snake families, similar response was reported for these molecules, from both venoms. Basic PLA_2_ induced the hydrolysis of membrane phospholipids and total cell hemolysis [80]. Despite the fact that acidic PLA_2_s are found abundantly in the snake venom, their role is poorly understood [81]. In spite of having high catalytic activity as compared to basic PLA_2_s, they do not induce toxicity [82]. Studies have suggested acidic PLA_2_s to participate in prey digestion [83]. Other studies have suggested that acidic PLA_2_s work synergistically with other venom toxins, as PLA_2_s, metalloprotease and cytotoxins [84,85,86].

PLA_2_ is ubiquitously found in nature [87,88]. In mammals, they are known to play important and vital role in many life processes [89,90,91]. On the other hand, snake venom PLA_2_s are toxic and interfere with a number of physiological processes, upon envenomation [87]. Phospholipase A_2_ are also known to be responsible for the hepatic injury, inflammation and anticoagulation in a victim [26].

#### 3.1.3. Snake Venom Metalloproteinase

The present study shows that *N. naja* and *N. oxiana* snake venom contain significant amounts of metalloproteinases, which are the second most abundant protein family. Proteomics study of other *Naja* species shows the presence of SVMPs in varying amounts ranging from as low as 0.9% to 16% [74,92,93,94,95,96,97,98,99,100]. Previous proteomic studies reported a lower abundance of SVMP in Pakistani *N. naja* venom [42,43,44]. Three SVMPs bearing relatively higher homology with snake venom metalloproteinase from *N. atra* were determined in each of the two venoms. Twenty fragments of SVMPs were detected in *N. naja* venom, which are homologous to K-like SVMPs from *N. atra*. 13 Peptide fragments were found to match with SVMPs from *N. kaouthia* (Table 2). The data shows that in case of *N. oxiana* venom higher number of peptide fragments match with SVMPs from *N. atra* venom (Table 3). The eastern Indian *N. naja* contains only 6% SVMPs in contrast to Pakistani *N. naja*, which contains 10% of SVMPS. It is interesting to note that western Indian *N. naja* contains 16% SVMPs as determined by A. K. Mukherjee and his research group [45,46].

SVMPs are found in all advanced snakes and make up the major component of the venom of Crotalid and Viperid snakes [101,102,103,104]. SVMPs are structurally versatile and act on different hemostatic targets of prey upon envenomation [105]. These toxins provoke many systemic changes, such as hemorrhage, acute renal failure, coagulopathy, and/or platelet aggregation inhibition [106]. The SVMPs identified in terms of our investigations, in both of the venoms, belong to subfamily P-III. The P-III SVMPs possess gelatinolytic and hemorrhagic activities [105]. A previous study reported the hemorrhagic response of Pakistani *N. naja* venom in chicken egg [107]. The determination of a higher amount of SVMPs in both NO and NN venom indicates that there is potential for hemorrhage as a response of NO and NN snakebite envenomation.

#### 3.1.4. L-Amino Acid Oxidase

Snake venom L-Amino acid oxidase (LAAOs) belong to the Flavin monoamine oxidase family and are dependent on FAD group for their activity. These proteins are present in both venoms studied and constitute approximately 4–5% of the venom proteome (Table 1). Peptide fragments bearing sequence similarity to LAAO from different snake venoms were detected and summarized (Table 2 and Table 3). In contrast to our results, studies of western Indian *N. naja* venom report only 0.31% LAAO. However, Indian *N. naja* venom contains 3% LAAO, which is similar to that of Pakistani *N. naja* [45,46]. In terms of our investigations we identified that LAAO from both, NN and NO venom, have homology with LAAO from *N. atra* venom. LAAO is known to be prevalent in many snake venoms [108] but its role in snake venom envenomation pathology is not clear. A recent study reported that LAAO might contribute to severe tissue disruption. This study suggested that LAAO might elicit its toxicity by catalyzing the intracellular substrates [108]. Different biological activities of the isolated LAAO have been reported like, edema, cytotoxic, antibacterial, antiparasitic, and/or platelet aggregation effects [109,110]. Also some investigations reported antitumor effects of LAAO [111]. LAAO is a glycoprotein and glycosylation is also considered to play a significant role in the toxicity of LAAO, and cause cell death by interacting with the cell surface [112,113].

#### 3.1.5. Cobra Venom Factor

Cobra venom factor (CVF) belong to the venom complement C_3_ homologue family. CVF constitutes approximately 9% of the total proteins identified in both venoms. The identified CVF peptides bear homology mainly to the CVFs from *N. kaouthia*. Fragments matching to CVF alpha chain and gamma chain were also analyzed. Peptide fragments showing sequence similarity to CVF proteins from other Elapidae and Colubridae have also been identified (Table 2 and Table 3). Proteomic study of Indian *N. naja* venom showed that it contains only 0.03–1.7% CVF [45,46], which is significantly less compared to our results obtained for the Pakistani *N. naja*. A venom proteome study of *Naja philippinensis* showed that it contains less than 4% [76]. The venom of *N. ashei* contains only 0.12% CVF [99]. Cobra venom factor is a complement activating protein and is functionally and structurally similar to complement component C3b. It is a glycoprotein and herein glycosylation contributes in the immunogenicity of CVF [114,115]. In vivo studies have shown that CVF causes an acute inflammatory injury in the lungs [116]. CVF serves as a gold standard molecule for the evaluation of drugs for trials, to control diseases involving the complement system [117]. A recent study reported CVF as a promising candidate for the treatment of IRI-induced hepatic injury [118]. Our data reveals that CVF is one of the abundant proteins in the venom of Pakistani *Naja naja* and *Naja oxiana* (Figure 3). Therefore, these venom can be a good source of isolating CVF for use in biomedical research.

#### 3.1.6. Cysteine-Rich Secretory Protein

Cysteine-rich secretory proteins (CRISPs) have been identified in many animal venoms. These proteins have two domains, a pathogenesis related domain at the N-terminal region and a cysteine rich domain at the C-terminus. Based on sequence homology the CRISP family is classified into four classes, and snake venom CRISPs belong to the group III [119]. CRISPs were found in much higher abundance in *N. naja* (7%) as compared to *N. oxiana* (2.8%) and peptide fragments showing similarities to CRISPs from different snake venoms were found in both venoms. However, highest similarity was found with the cysteine-rich venom protein natrin-1(NA-CRVP1) from *N. atra*. Investigations indicated that NA-CRVP1 could act as inflammatory modulator that could perturb the wound-healing process of a bitten victim by regulating the expression of adhesion molecules in endothelial cells. This study also showed that natrin contains a zinc-binding domain at the N-terminus and elicits its proinflammatory activity in a zinc and heparan-sulfate dependent manner [119]. Natrin has also been reported as a potassium channel blocker and in this context can weakly block muscle contraction [120,121,122,123]. In our study six peptide fragments matching CRISP from *N. haje*. A study reported this CRISP was found to be non-toxic when administered to crickets [124]. The venom proteome of *N. haje* contain 10% CRISP [92]. Different species of *Naja* contain varying amounts of CRISP, from as low as 0.2% to 10% of the total venom proteome. The Indian *N. naja* contains 1.14–3% CRISPs [45,46].

#### 3.1.7. Snake Venom Serine Proteinase

Snake Venom Serine Proteinase (SVSPs) belong to the peptidase S1 family. *N. oxiana* venom proteome shows relatively higher abundance of serine proteinases (4%) as compared to *N. naja* venom, which contains only 2% (Table 1; Figure 3). Both of the venoms contain peptide fragments, which bear homology to tissue-type plasminogen activators from *Ophiophagus hannah* and the thrombin-like enzyme TLP from Indian *N. naja.* In addition to this, peptide fragments having sequence similarity to SVSP have also been identified (Table 2 and Table 3). SVSPs have been identified in only few *Naja* species venom. In western Indian *N. naja* the SVSPs contributed only 0.69% to venom proteome [46]. *N. philippinensis* venom proteome consists of 0.35% SVSPs [76]. Previous studies showed that SVSPs are absent in Eastern Indian *N.naja* venom, while a small percentage (0.03%) was reported for the western Indian snake [45,46].

SVSPs have high specificity towards their substrates. Based on their biological roles, they have also been classified as activators of the fibrinolytic system, procoagulant, anticoagulant and platelet-aggregating enzymes [125]. A few SVSPs, like ancrod and batroxobin have already applications in the treatment of cardiovascular problems, while reptilase serves today as a diagnostic probe for dysfibrinogenemia [126].

#### 3.1.8. Snake Venom Nerve Growth Factor

Snake venom Nerve Growth Factor (NGF) were identified in both venoms but were relatively more abundant in the venom of *N. oxiana* (4%) as compared to *N. naja* (2%), Table 3. In both venoms, peptides sharing homology with *Ovophis okinavensis, N. sputatrix,* and *Bitis gabonica* NGF were identified (Table 1 and Table 2). In *N. naja* seven peptides bearing homology with *Pseudechis australis* were also identified. Further, additional peptide fragments of NGF were also identified in terms of our investigations (Table 2 and Table 3). A previous proteomic study also showed Pakistani *N. naja* venom to contain 2% NGF [42]. *N. naja* snake venom from east India contained 3.1% and 1% in western India sample. In the same study *N. kaouthia* from eastern India was shown to contain 1% NGF [45,46]. *N. philippinensis* contain only 0.06% NGF [76]. Proteomic analysis of other *Naja* species venom have also shown them to contain NGF but their relative abundance was not calculated [93,100]. Moroccan cobra venom contains 5% NGF of total venom proteome [92].

Till now not much is known about the contribution and function of NGFs in envenomation. Various bioassays have shown that NGFs have neurotropic activity. Snake venom NGFs have been suggested as a pharmacological tool to study the structure function relationship of human trkA receptor [127]. Studies show that NGFs assert venom toxicity indirectly, either by acting as a carrier of other neurotoxins, which do not have specific recognition sites, like phospholipase or by inducing plasma extravasation at the site of snakebite. NGF is known to coexist with CVF in cobra snake venom, and might be responsible for enhancing the toxic mechanism of CVF in an indirect manner [128]. In 1986, two scientists were awarded a Nobel Prize for their pioneering work, which allowed to explain cell growth regulation. And in context of this investigations Cohen serendipitously discovered NGF from snake venom of *Agkistrodon piscivorus* [129].

#### 3.1.9. Snake Venom Phosphodiesterase

A lower abundance of snake venom phosphodieterases (PDEs) was determined in both venoms, although relatively higher in *N. oxiana,* i.e., 3.1%. *N. naja* venom contains only 1.1% of PDEs. Peptides fragments matching with PDEs from the venom of *N. atra, Ovophis okinavensis,* and *Borikenophis portoricensis* were identified in both venom. A recent study determined PDE activity in the venom of ten different species of *Naja.* All the species showed PDE activity with minor variation [73]. The Indian *N. naja* venom was reported to contain less than 1 % PDEs, which is similar to Pakistani *N. naja.*

PDEs are ubiquitously present in snake venom but their activity is higher in Viperidae venom as compared to Elapidae family [130]. In recent years, there has been considerable interest in snake venom PDEs due to their potential applications as pharmacological tool and drug lead. The endonuclease activity of PDEs rendered their use in sequencing of polynucleotides and oligonucleotides [130]. Phosphoribosylation, a protein modification, can also be processed by PDEs [131]. Recent innovative approaches, have utilized snake venom PDEs to digest genomic DNA into single nucleosides to study modifications of DNA [132,133,134].

### 3.2. Minor Protein Components (Relative Abundance ≤2%)

A large number of low abundant proteins were found in both venoms, particularly in *N. naja* (Table 2 and Table 3). Ras-like proteins, identified in the venom of *N. naja* were of particular interest, as they indicate the presence of extra0cellular vesicles in the venom. Snake venom extracellular vesicles (SVEVs) have previously been isolated from the venom of *Agkistrodon contortrix contortrix*, *Crotalus atrox*, *Crotalus viridis,* and *Crotalus cerberus oreganus*. The size distribution of SVEVs was found to be between approximately 50–500 nm. Proteomic investigations revealed that SVEVs could be assigned to eight different protein classes, such as SVMP, SVSP, and disintegrins [135].

Exosome-like vesicles have also been reported in the venom of *Gloydius blomhoffii blomhoffii* [136]. In this context extracellular vesicles (EV) are known to carry a diverse cargo of molecules as proteins, DNA, RNA, and/or lipids [137]. Further, EVs are involved in cell-to-cell communication, immune response and apoptotic rescue [138,139] and participate in the maintenance of normal as well as pathophysiological conditions, like cancer [140,141,142]. The proteomic study of extracellular vesicles released from cancer cells have shown the presence of Ras proteins functioning as biomarkers for extracellular vesicles [137,143,144,145]. Studies have shown that Ras proteins are involved in the regulation and assembly of extracellular vesicles cargo [145,146,147,148]. Therefore, the identification of Ras-like proteins indicates the presence of extracellular vesicles in the venom of *N. naja*. However direct experimental work needs to be done to confirm the presence of such vesicles in the venom. SVEVs in the venom may be involved in another mechanism to secrete membrane proteins like aminopeptidase A and G coupled receptors. SVEVs may also offer an additional route for the envenomation process, thereby facilitating toxins to translocate inside the prey cells.

In the present work, a number of proteins have been identified for the first time in the proteome of these venoms, like G-protein coupled receptors, zinc finger proteins, ankyrin repeat, leucine repeat, Ubiquitin carboxyl-terminal hydrolase and a number of other protein. It can be assumed that these proteins have also a function in the venom. Ankyrin repeats and zinc finger proteins were also identified recently, in the venom of King cobra, *Naja annulifera* and *Micrurus pyrrhocryptus* [51,100,149]. A rather old publication reported cobra serum albumin in the venom of cobra snakes [150]. Our data also revealed the presence of cobra serum albumin in the venom of *N. naja.* It is possible that upon envenomation cobra serum albumin is responsible or supporting the transportation of other venom proteins in the prey serum. Previous studies have reported Cobra blood serum albumin as an antitoxic protein, having the potential to sequester endogenous toxins [151,152]. Cobra serum albumin was also reported in the venom proteome of *N. sumatrana* [93]. Further, we identified glutathione peroxidase in both venoms. A recent proteomic study also reported the presence of glutathione peroxidase in the venom of *Micrurus pyrrhocryptus* and *N. annulifera* [100,149]. It can be speculated that this protein might be involved in protecting the venom gland from oxidative damage. Phospholipase A_2_ inhibitors, bearing similarity to PLA_2_ inhibitor isolated from the serum of *Elaphe quadrivirgata and Naja kaouthia* snakes, were identified in the venom of *N. naja.* This inhibitor was shown to inhibit the enzymatic activity of all till now known PLA_2_ enzymes [153,154]. Phospholipase B was also identified in both venom. Only 0.1% constituted the venom proteome of NN while that of NO contained 1.6% of the total venom proteome. Studies have shown that PLBs make up approximately 0.34% of venom components, and in Viperidae venom it varies between 0.23% to 2.5% [155]. Insulin and Transferrin proteins were also identified in the venom of *N. naja.* Transferrin is a metal binding proteins. Transferrin was also reported before in the venom of *P. australis*, utilizing two dimensional gel electrophoresis [156]. Snake venom VEGF bearing similarities to that isolated also in *Bitis arietans* venom, identified in *N. naja* venom as well. Studies have shown that different variants of snake venom VEGF induce angiogenesis and vascular permeability through different mechanisms [157,158]. Snake venom VEGF are potential candidates for therapeutic angiogenesis [159]. A low abundance of Kunitz type serine protease inhibitors (KSPI) was identified in the venom of both snakes. Snake venom KSPI have the potential to selective inhibit distinct serine proteases [35]. Some of the snake venom KSPI have evolved as potassium channel blockers [160]. BF9 a snake venom KSPI, which act as potassium channel blockers and retain the serine protease inhibitory activity. This bifunctional KSPI was reported in the venom of *Bungarus fasciatus* [161]. Interestingly another type of serine protease inhibitor, i.e., serpin, was identified in the venom of *N. oxiana*. Proteins belonging to Ohanin/Vespryn family were found in both venoms. They are small proteins with an average mass of approx. 12 kDa, and are neurotoxic in nature [162]. Further, we could identify 5′-nucleotidase in both venoms. This family of protein is found in different snake venoms [163]. These enzymes play a major role in the release of adenosine upon envenomation, which facilitates prey immobilization. In vivo studies have shown that 5-nucleotidases act synergistically with other venom components like phospholipases, disintegrins to exert a pronounced anticoagulant effect [164]. Aminopeptidase was identified in both *N. naja* and *N. oxiana* venoms. Aminopeptidase A activity has been found in the venoms of snakes belonging to Elapidae and Viperidae families. Till now not much is known about the contribution of this enzymes within envenomation pathology [165]. Cystatin, having similarity to cystatin from the venom of *N. kaouthia,* was identified for the first time in the venom of *N. naja* in the present study. Cystatin is a cysteine protease inhibitor [166]. Natriuretic peptides were identified in both *N. naja* and *N. oxiana* venom. These peptides are known to induce hypotension upon envenomation [167,168]. Cathelicidin was identified in the venom of *N. naja*, and previous studies have shown it to be potent antimicrobial peptide [169].

### 3.3. Posttranslational Modifications

In terms of our investigations, we were able to identify N-terminal acetylation (N-ace) for the first time in the snake venom. This posttranslational modification is known to carry and support out different functions in the cell. A most analyzed function of N-ace is the regulation of protein half-life, by labelling proteins for polyubiquitation and thus degradation by the proteasome [170,171]. N-ace modification plays a role in protein folding and protein complex formation [172,173]. Furthermore, studies have shown that N-ace modification mediates the interaction of proteins with membrane and subcellular localization [173]. A probable role of this modification in snake venom proteins could be to stabilize them against proteolytic cleavage, and to assist in distinct protein–protein interactions upon envenomation. In both venoms a peptide fragment bearing homology to muscarinic toxin like-protein 3, from the venom of *Naja kaouthia* was found to be N- terminal acetylated. Whereas in *Naja naja* two other peptide fragments were identified to be N-terminal acetylated. One bearing homology to phospholipase A_2_ and other to a weak neurotoxin 7 (Supplementary Table S1 and S2).

## 4. Conclusions

Using the MS shotgun approach we could provide a holistic view of the venom profile of the two Pakistani cobra snakes *N. naja* and *N. oxiana*. Our data shows for the first time the venom proteome of *N. oxiana*. The comparative evaluation of the venom proteome of the two snakes reveals differences, as well as similarities in their venom composition. Both snake venoms contain different types of three-finger toxins in their venom, although they exit sympatrically. There are a few toxin families, which were only found in the venom of *N. naja*, like cystatin, VEGF, TGF, BPP, and Cathelicidin. Therefore, we can suggest, that venom samples from both species should be utilized for the production of effective antivenoms. Also, applying state-of-the-art mass spectrometric tools allowed to identify a number of proteins not known before to be in these venoms, like Ras-GTPase, Ankyrin repeats, leucine repeat, G-protein coupled receptor, zinc finger protein, holiday junction protein, and endonuclease. In this context, identification of Ras-like proteins provided a clue about the presence of extracellular vesicles. These vesicles might function as an additional carrier to transport venom components in the prey upon envenomation. Further, our data highlight N-terminal acetylation of venom proteins for the first time and the results delineate the unique complexity of snake venoms, and open routes for further research to understand function of these molecules upon envenomation.

## 5. Materials and Methods

### 5.1. Venom Collection

Venom was milked manually from adult snake species of *N. naja* (black cobra/Indian cobra/Spectacled cobra) and *N. oxiana* (brown cobra/Caspian cobra/Central Asian cobra). For the proteomic studies of each species the venom was collected from three adult healthy snakes and pooled. The sex of the snakes was not determined. *N. naja* snakes were captured from the rural surroundings of Mianwali district, while *N. oxiana* snakes were caught from Lahore, Punjab province, Pakistan. The venom was freeze dried and kept at −20 °C till further use.

### 5.2. Sample Preparation for LC-MS/MS

For LC-MS/MS analysis the lyophilized crude venom from *N. naja* (black cobra) and *N. oxiana* (brown cobra) was dissolved in 10 mM Triethylamonium bicarbonate (TEAB, Thermo Fisher Scientific), 1% *v/w* Sodium deoxycholate (SDC, Sigma) buffer. Protein concentration was determined using a bicinchoninic acid protein assay (Pierce™ BCA Protein Assay Kit, Thermo Fisher Scientific) and 20 µg of venom protein was tryptically digested. In brief, cysteine residues were reduced for 30 min. in the presence of 10 mM dithiothreitol (DTT, Sigma) at 60 °C and alkylated for 30 min. with 20 mM iodoacetamide (IAA, Sigma) at 37 °C in the dark. Thereafter, sequencing grade trypsin (Promega) was added in a protease/protein ratio of 1:100 at 37 °C to hydrolyze venom proteins overnight. Enzymatic activity was quenched by addition of 1% *v/v* formic acid (FA, Fluka) and the SDC was precipitated by centrifugation at 16000 g for 5 min. The peptide containing supernatant was vacuum dried and reconstituted in 0.1% FA for LC-MS/MS analysis.

### 5.3. LC-MS/MS Analysis of the Digested Venom

LC-MS/MS analysis of the venom samples was performed using a nano ACQUITY UPLC^®^ System (Waters, Manchester, UK) coupled to a Hybrid-Quadrupole-Orbitrap mass spectrometer (Q Exactive™, Thermo Fisher Scientific). The LC system was equipped with a reversed phase chromatography (RPC) columns [ACQUITY UPLC^®^ Symmetry C 18 (180 µm i.d × 20 cm, 5 µm particle size, 100 Å pore size, Waters, Manchester, UK) as trap column and a RPC separation column (ACQUITY UPLC^®^ Peptide BEH C-18 (75µm i.d × 20 cm, 1.7 µm particle size, 170 Å pore size, Waters, Manchester, UK) as analytical column. RPC was used with a linear 60 min acetonitrile gradient from 2–30% for peptide separation. (Solvent A: 0.1% FA in water; Solvent B: 0.1% FA in acetonitrile; Flow rate of 250 nL/min).

MS/MS data acquisition was performed in data dependent mode for the 15 most abundant precursor ions. Precursor ions with charge stages between 2+ and 5+ were selected for fragmentation if they exceeded an intensity threshold of 100,000. For MS/MS spectra acquisition the set AGC-target was 100,000 with a maximal ion injection time of 50 ms. Precursor ions were fragmented at a normalized collision energy (NCE) of 25 and the fragment ions were measured with a resolution of 17,500 at 200 m/z. To avoid redundant precursor sampling a dynamic exclusion was applied for 20 s.

### 5.4. Data Analysis

For protein identification, the generated raw data were processed using the Proteome Discoverer™ Software 2.0.0.802. Database search was performed with the SEQUEST algorithm against an *Ophiophagus hannah* (txid:8665, King cobra) protein database (UniProt), since it represents the closest sequence database to the analyzed samples. Carbamidomethylation of cysteine was used as fixed modification. Furthermore, oxidation of methionine, conversion of glutamine to pyro-glutamic acid at peptide N-termini, loss of N-terminal methionine and the acetylation of protein N-termini were considered as variable modifications. Precursor and fragment ion tolerance were set at 10 ppm and 0.02 Da, respectively. Peptide-spectra matches with a maximum delta Cn of 0.05 were used by Percolator for FDR estimation.

Unidentified spectra were exported to a new mgf file and de novo sequencing was performed with Novor [174] via DenovoGUI 1.16.2 [175]. Modifications and allowed mass tolerances were identical to the database search approach. Hits with a Novor score above 80 were considered for a protein BLAST approach. Protein BLAST for the sequenced peptides was conducted with the NCBI BLAST p algorithm (2.9.0+) with default settings against non-redundant protein sequences (nr) narrowed down to serpents (taxid: 8570). Alignments were chosen according to the max Score, the query coverage and if the homologous proteins were related to venom activity. With this information, a venom specific peptide database was created to support database searching for further analyses. Similarly, the data search was also performed against Serpents protein data base from UniProt.

The mass spectrometry proteomics data have been deposited to the ProteomeXchange Consortium via the PRIDE partner repository [176] with the dataset identifier PXD018726 and 10.6019/PXD018726. 

Venom components were classified according to protein families and their relative abundances calculated as, reported previously [51]. Briefly, the proteins analyzed were sorted into different groups of protein families. The relative abundance of each family was calculated as percent of total number of venom proteins detected by the mass spectrometer. The mathematical relationship below was used to calculate the relative abundance of each protein group. Pie chart (Figure 3) and Table 1, presents the percentage relative abundance of proteins.

(1) $$\frac{\mathrm{Number}\;\mathrm{of}\;\mathrm{proteins}\;\left(\mathrm{protein}\;\mathrm{family}\right)\;}{\mathrm{Total}\;\mathrm{venom}\;\mathrm{proteins}\;\mathrm{detected}\;\mathrm{using}\;\mathrm{LC}-\mathrm{MS}/\mathrm{MS}}\;\times \;100$$

## Figures and Tables

**Table 1:** Comparative evaluation of snake venom protein families in the venom of *N. naja* and *N. oxiana.*

Protein Family	First Report in Nn Venom	Abbreviation Used	NN (No of Peptides)	%Age	NO(No. of Peptides)	%Age
Three-Finger toxin		3FTX	157	21	41	16
Snake venom metalloprotease family		SVMP	72	10	39	15
Cobra venom factor		CVF	62	9	22	8.7
Cysteine-rich secretory protein		CRISP	53	7	7	2.8
Phospholipase A2		PLA2	46	6	32	12.6
Phospholipase B		PLB	1	0.1	4	1.6
**Phospholipase inhibitor**	✓	CNF-I	3	0.4	-	-
L-amino-acid oxidase		LAAO	31	4	14	5.5
Snake Venom Serine proteinase		SP	15	2	11	4.3
Ohanin		Oh	11	1.5	2	0.8
Kunitz type serine protease inhibitor		KSPI	14	2	4	1.6
Nerve Growth Factor		NGF	12	1.7	11	4.3
5′-nucleotidase		5-Ntd	10	1.4	1	0.4
**Serum Albumin**	✓	SA	10	1.4	-	
Glutathione peroxidase	✓	GP	9	1.2	3	1.2
Phosphodiesterase		Pde	8	1.1	8	3.1
Aminopeptidase		-	7	1	4	1.6
TNF receptor family	✓	TNF	2	0.3	3	1.2
Lectin	✓	-	3	0.4	1	0.4
Natriuretic peptide family		NP	4	0.54	1	0.4
Cystatin		-	4	0.54	-	
Cathelicidin	✓	cath	1	0.1	-	
N-acetylcholinesterase		N-Ache	1	0.1	1	0.4
**Vascular endothelial growth factor**		VEGF	1	0.1	-	
**Transforming growth factor**	✓	TGF	2	0.3	-	
Zinc finger protein	✓	ZFP	6	0.8	4	1.6
**Insulin**	✓	In	2	0.3	-	
**Transferrin**	✓	TF	2	0.3	-	
Ankyrin repeat	✓	AR	2	0.3	1	0.4
Leucine repeat	✓	LR	1	0.1	1	0.4
**Endonuclease**	✓	-	3	0.4	-	
SLRP family	✓	SLRP	2	0.3	1	0.4
**Ras-like protein**	✓	Ras	5	0.7	-	
Serpin		-	-		1	0.4
Others		-	158		37	
Total			735		254	

Bold text in the first column indicates protein families exclusively identified in N. naja venom. Blue coloured text indicates protein family identified only in N. oxiana. Check mark (✓) in the second column, indicates that this work is the first report of the identification of the corresponding protein families in N.naja venom. The dash sign indicate that the protein family was not identified in the venom.

**Table 2:** Summary of the venom proteome of *Naja naja*.

S. No	Protein Family	Protein	Accession Code	Number of Matched Peptides	Homology with a Protein from the Venom of Snake Species
1	3FTXs (Neurotoxin)	Long neurotoxin	AHZ08824	9	*Micropechis ikaheca*
2			P01389	1	*Naja anchietae*
3			P01390	2	*Naja nivea*
4		Long neurotoxin homolog	O93422	5	*Naja atra*
5		Long neurotoxin 1	P25668	4	*Naja naja*
6		Long neurotoxin 1	P01380	1	*Hydrophis stokesii*
7		Long neurotoxin 1	P25674	3	*Naja haje haje*
8		Long neurotoxin 4	P25672	3	*Naja naja*
9		Long neurotoxin 7	O42257	3	*Naja sputatrix*
10		putative long neurotoxin	ABX58151	1	*Austrelaps labialis*
11		putative long neurotoxin	ABX58163	1	*Austrelaps labialis*
12		Alpha-neurotoxin NTX-3	O57326	1	*Naja sputatrix*
13		Short neurotoxin 3	P01420	1	*Naja annulifera*
14		Short neurotoxin III	P59275	1	*Naja kaouthia*
15		Neurotoxin II	P01427	6	*Naja oxiana*
16		cobrotoxin b	CAA73829	3	*Naja atra*
17		Cobrotoxin-b	P80958	4	*Naja atra*
18		Alpha-cobratoxin	P01391	4	*Naja kaouthia*
19		kappa-cobrotoxin	CAA76846	1	*Naja atra*
20		Weak toxin 2	Q8AY50	2	*Bungarus candidus*
**21**		**Weak neurotoxin 7**	**P29181**	**7**	***Naja naja***
22		Weak neurotoxin 10	Q802B2	1	*Naja sputatrix*
23		Weak toxin CM-11	P01401	4	*Naja haje haje*
24		Weak toxin S4C11	P01400	5	*Naja melanoleuca*
25		three-finger toxin precursor, partial	ADN67572	4	*Bungarus multicinctus*
26		three-finger toxin precursor, partial	ADN67582	9	*Naja atra*
27		three-finger toxin precursor, partial	ADN67583	1	*Naja atra*
28		three-finger toxin precursor	ADN67579	1	*Naja atra*
**29**		**Muscarinic toxin-like protein 3**	**P82464**	**3**	***Naja kaouthia***
30		Muscarinic toxin-like protein	Q9W727	4	*Bungarus multicinctus*
31		Muscarinic toxin-like protein 2	P82463	6	*Naja kaouthia*
32		Muscarinic toxin-like protein 1	P82462	2	*Naja kaouthia*
33		Muscarinic toxin 38	Q2VBN0	1	*Ophiophagus hannah*
34		Alpha-elapitoxin-Nk2a	P01391	4	*Naja kaouthia*
36		three finger toxin V	ABX82866	1	*Walterinnesia aegyptia*
37		Three finger toxin W-V	C1IC49	3	*Walterinnesia aegyptia*
38		Chain A, Putative Ancestral Mamba Toxin 1	5MG9_A	1	*Dendroaspis angusticeps*
39	3FTXs (cytotoxins)	cytotoxin 17, partial	BAU24676	13	*Naja naja*
40		Cytotoxin Vc-5	Q9PS34	6	*Naja oxiana*
41		Cytotoxin 3a	P86539	4	*Naja naja*
42		Cytotoxin SP15c	P60308	13	*Naja atra*
43		cardiotoxin 7a	AAB36929	2	*Naja atra*
44		cardiotoxin 7a	Q91126	3	*Naja atra*
46		Cytotoxin 8	P86540	2	*Naja naja*
47		Cytotoxin 1	P01447	1	*Naja naja*
48		Cytotoxin II	P01441	1	*Naja oxiana*
49		Cytotoxin 5	P25517	2	*Naja mossambica*
50		Cardiotoxin-6	Q98965	1	*Naja atra*
51		Cytotoxin 10	P86541	1	*Naja naja*
52		Cytotoxin homolog 3	P01473	1	*Naja melanoleuca*
53		Cardiotoxin-like basic polypeptide ah	P0C547	2	*Naja atra*
54		cardiotoxin 1e	AAA90960	4	*Naja atra*
55	Venom complement C_3_-like	Venom factor	AAX86641	5	*Austrelaps superbus*
56		Cobra venom factor	Q91132	31	*Naja kaouthia*
57		Cobra venom factor gamma chain	Q91132	2	*Naja kaouthia*
58		Cobra venom factor alpha chain	Q91132	2	*Naja kaouthia*
59		cobra venom factor precursor	AAA68989	1	*Naja kaouthia*
60		venom factor-like, partial	XP_025025833	2	*Python bivittatus*
61		cobra venom factor 1, partial	AXL96620	13	*Ahaetulla prasina*
62		cobra venom factor, partial	AXL95279	1	*Spilotes sulphureus*
63		cobra venom factor, partial	AWX67646	1	*Boiga irregularis*
64		Ophiophagus venom factor	I2C090	3	*Ophiophagus hannah*
66	Venom Kunitz-type family	Kunitz-type serine protease inhibitor	P19859	1	*Naja naja*
67		Kunitz-type serine protease inhibitor	P20229	6	*Naja naja*
68		Kunitz-type serine protease inhibitor isoform 7	ACY68703	1	*Parasuta nigriceps*
69		Kunitz inhibitor b, partial	AAL30069	1	*Bungarus candidus*
70		protease inhibitor	AFA90080	1	*Daboia siamensis*
71		Venom basic protease inhibitor 2	P00986	1	*Naja nivea*
72		Kunitz-type protease inhibitor, partial	AWX67660	1	*Boiga irregularis*
73		papilin-like, partial	XP_025032351	1	*Python bivittatus*
74		Kunitz inhibitor I	ABX82867	1	*Walterinnesia aegyptia*
75	natriuretic peptide family	Natriuretic peptide Na-NP	D9IX97	2	*Naja atra*
76		natriuretic peptide	ADK12001	1	*Naja atra*
77		natriuretic peptide	ADK12001	1	*Naja atra*
78	cystatin	Cystatin	E3P6P4	4	*Naja kaouthia*
79	NGF-beta family	Venom nerve growth factor 2	Q5YF89	2	*Naja sputatrix*
80		Venom nerve growth factor 3	Q3HXY1	7	*Pseudechis australis*
81		nerve growth factor, partial	AAR24530	1	*Bitis gabonica*
82		nerve growth factor	BAN82142	4	*Ovophis okinavensis*
83		nerve growth factor beta chain precursor	A59218	1	*Naja kaouthia*
84	Ohanin/vespryn family.	Ohanin	P83234	4	*Ophiophagus hannah*
85		Thaicobrin	P82885	2	*Naja kaouthia*
86		Venom PRY-SPRY domain-containing protein, partial	AHZ08803	4	*Micropechis ikaheca*
87		Vespryn	AEJ32004	1	*Crotalus adamanteus*
88	Insulin family	Insulin-like growth factor-binding protein 3, partial	XP_025032248	1	*Python bivittatus*
89		Insulin enhancer protein ISL-1, partial	ETE72105	1	*Ophiophagus hannah*
90	Snake venom VEGF subfamily	Snake venom vascular endothelial growth factor toxin barietin	C0K3N1	1	*Bitis arietans*
91	CRISP	Cysteine-rich venom protein 25	P84806	6	*Naja haje haje*
92		cysteine-rich seceretory protein Ts-CRPM	ACE73574	2	*Trimeresurus stejnegeri*
93		Cysteine-rich venom protein mossambin	P0DL16	2	*Naja mossambica*
94		Cysteine-rich venom protein natrin-1	Q7T1K6	16	*Naja atra*
95		Cysteine-rich venom protein ophanin	Q7ZT98	3	*Ophiophagus hannah*
96		cysteine-rich venom protein, partial	BAP39957	1	*Protobothrops flavoviridis*
97		Cysteine-rich venom protein natrin-2	Q7ZZN8	3	*Naja atra*
98		Cysteine-rich seceretory protein Ts-CRPM	N-ACE73574	1	*Trimeresurus stejnegeri*
99		Cysteine-rich venom protein 25-A	P84807	1	*Naja haje haje*
100		Helicopsin	P0DJG8	2	*Helicops angulatus*
101		Cysteine-rich venom protein bucarin	P81993	1	*Bungarus candidus*
102		Cysteine-rich venom protein latisemin	Q8JI38	1	*Laticauda semifasciata*
103		Cysteine-rich venom protein ophanin	AAO62996	1	*Ophiophagus hannah*
104		cysteine-rich secretory protein 4, partial	AXL96584	2	*Borikenophis portoricensis*
105		Cysteine-rich venom protein kaouthin-1	P84805	1	*Naja kaouthia*
106		Cysteine-rich venom protein annuliferin-b	P0DL15	1	*Naja annulifera*
107		Cysteine-rich venom protein	AAP20603	2	*Naja atra*
108		Cysteine-rich secretory protein	AJB84505	1	*Philodryas chamissonis*
109		Opharin precursor	AAP81292	1	*Ophiophagus hannah*
110		Cysteine rich secretory protein 2, partial	AXL96629	4	*Ahaetulla prasina*
111	Cathelicidin family	Cathelicidin-related protein precursor	ACF21000	1	*Naja atra*
112	TGF-beta family	Transforming growth factor beta-3, partial	ETE71774	1	*Ophiophagus hannah*
113		Glial cell line-derived neurotrophic factor, partial	ETE67324	1	*Ophiophagus hannah*
**114**	**Phospholipase A2**	**Acidic phospholipase A2 3**	**P60045**	**4**	***Naja sagittifera***
115		85 kDa calcium-independent phospholipase A2, partial	ETE71158	2	*Ophiophagus hannah*
116		Acidic phospholipase A2 1	P00596	4	*Naja kaouthia*
117		Acidic phospholipase A2 1	Q9W7J4	6	*Pseudonaja textilis*
118		Basic phospholipase A2 T1-2 A chain	P84472	2	*Bungarus candidus*
119		Acidic phospholipase A2 C	Q92086	5	*Naja sputatrix*
120		Acidic phospholipase A2 1	P00598	3	*Naja naja*
121		Acidic phospholipase A2 2	P60044	1	*Naja sagittifera*
122		Acidic phospholipase A2 1	P00596	4	*Naja kaouthia*
123		Phospholipase A2	BAA36403	1	*Naja kaouthia*
124		Acidic phospholipase A2 beta-bungarotoxin A4 chain	P17934	2	*Bungarus multicinctus*
125		Phospholipase A2-III	ABD24038	1	*Daboia russelii russelii*
126		Basic phospholipase A2 homolog 1	P10117	1	*Laticauda colubrina*
127		Phospholipase A2	AAL55555	1	*Hydrophis hardwickii*
128		Phospholipase A2	P15445 (2WQ5)	1	*Naja naja*
129		Phospholipase A2 3	P21792	3	*Micrurus nigrocinctus*
130		Phospholipase A2I precursor	BAC77655	1	*Bungarus flaviceps*
131		Phospholipase a2	CAA45372	1	*Naja naja*
132		Phospholipase A2	AAA66029	1	*Naja naja*
133		Phosphatidylcholine 2-acylhydrolase T1-2 A	P84472	2	*Bungarus candidus*
134	Phospholipase B-like family	Phospholipase B-like 1, partial	ETE59578	1	*Ophiophagus hannah*
135	CNF-like-inhibitor family	Phospholipase A2 inhibitor subunit gamma A	Q9PWI4	1	*Elaphe quadrivirgata*
136		Phospholipase A2 inhibitor beta subunit isoform OMI-2B	AAF21049	1	*Oxyuranus microlepidotus*
137		Phospholipase A2 inhibitor 31 kDa subunit	Q7LZI1	1	*Naja kaouthia*
138	SVMP (PIII)	Acutolysin e precursor	AAD27891	1	*Deinagkistrodon acutus*
139		Snake venom metalloproteinase	D5LMJ3	12	*Naja atra*
140		Snake venom metalloproteinase	D3TTC1	20	*Naja atra*
141		Snake venom metalloproteinase	D3TTC2	8	*Naja atra*
142		Snake venom metalloproteinase-disintegrin-like mocarhagin	Q10749	7	*Naja mossambica*
143		Snake venom metalloproteinase	Q9PVK7	5	*Naja kaouthia*
144		Snake venom metalloproteinase	A8QL49	2	*Bungarus multicinctus*
145		Snake venom metalloproteinase	P82942	8	*Naja kaouthia*
146		Snake venom metalloprotease(ADAM)	ACS74986	1	*Philodryas olfersii*
147		Snake venom metalloproteinase 27, partial	AXL96577	1	*Borikenophis portoricensis*
148		Disintegrin and metalloproteinase domain-containing protein 21, partial	ETE71596	2	*Ophiophagus hannah*
149		Microlepidotease-1	ABQ01137	1	*Oxyuranus microlepidotus*
150		Metalloproteinase atrase B, partial	ADD14036	1	*Naja atra*
151		Metalloproteinase 7, partial	AXL96626	1	*Ahaetulla prasina*
152		Snake venom metalloproteinase	P0DM46	1	*Micrurus corallinus*
153		K-like metalloprotease precursor, partial	ACN50005	1	*Naja atra*
154	Snake venom serine proteinase (peptidase S1 family)	Tissue-type plasminogen activator, partial	ETE66683	3	*Ophiophagus hannah*
155		Tissue-type plasminogen activator-like, partial	XP_025033187	3	*Python bivittatus*
156		Complement factor B precursor	AAR21601	1	*Naja kaouthia*
157		Thrombin-like enzyme TLP	P86545	2	*Naja naja*
158		Serine endopeptidase	AUS82567	1	*Crotalus tigris*
159		Snake venom serine protease NaSP	A8QL53	1	*Naja atra*
160		Snake venom serine protease catroxase-1	Q8QHK3	1	*Crotalus atrox*
161		Anionic trypsin-1-like	XP_007434941	1	*Python bivittatus*
162		Coagulation factor X isoform 1, partial	ETE73401	1	*Ophiophagus hannah*
163		Serine endopeptidase	AUS82552	1	*Crotalus scutulatus*
164	5’-nucleotidase family	5-nucleotidase	BAP39972	5	*Protobothrops flavoviridis*
165		Venom 5’-nucleotidase	A0A2I4HXH5	3	*Naja atra*
166		5’-nucleotidase, partial	ETE67245	1	*Ophiophagus hannah*
167		Snake venom 5’-nucleotidase	B6EWW8	1	*Gloydius brevicaudus*
168	Aminopeptidase	Aminopeptidase N, partial	ETE61021	1	*Ophiophagus hannah*
169		Aminopeptidase N	BAG82599	6	*Gloydius brevicaudus*
170	Type-B carboxylesterase/lipase	N-acetylcholinesterase	AAC59905	1	*Bungarus fasciatus*
171	Phosphodiesterase	Snake venom Phosphodiesterase	A0A2D0TC04	3	*Naja atra*
172		Phosphodiesterase	AHJ80885	1	*Macrovipera lebetina*
173		Phosphodiesterase, partial	AXL96599	2	*Borikenophis portoricensis*
174		Phosphodiesterase	BAN89425	2	*Ovophis okinavensis*
175	Flavin monoamine oxidase family	L-amino acid oxidase, partial	AAZ08620	1	*Daboia siamensis*
176		L-amino acid oxidase, partial	AVX27607	4	*Naja atra*
177		L-amino-acid oxidase	Q4JHE1	5	*Pseudechis australis*
178		L-amino-acid oxidase	P0C2D5	1	*Protobothrops flavoviridis*
179		L-amino-acid oxidase	A8QL51	1	*Bungarus multicinctus*
180		L-amino-acid oxidase	Q4JHE3	3	*Oxyuranus scutellatus scutellatus*
181		L-amino acid oxidase, partial	AVX27607	4	*Naja atra*
182		L-amino-acid oxidase	A8QL58	6	*Naja atra*
183		L-amino-acid oxidase	Q4JHE3	3	*Oxyuranus scutellatus scutellatus*
184		L-amino acid oxidase precursor	AAY89682	2	*Pseudechis australis*
185		L-amino-acid oxidase	CAQ72894	1	*Echis ocellatus*
186	True venom lectin family	C-type lectin galactose-binding isoform	D2YVK1	2	*Hoplocephalus stephensii*
187		BJcuL precursor	AAQ92957	1	*Bothrops jararacussu*
188	Ankyrin SOCS box (ASB) family	Ankyrin repeat and SOCS box protein 7, partial	ETE63895	1	*Ophiophagus hannah*
189		Ankyrin repeat domain-containing protein 50, partial	ETE61041	1	*Ophiophagus hannah*
190	Transferrin	Transferrin	CAK18221	2	*Natrix natrix*
191	Cobra serum albumin	Cobra serum albumin	S59517	1	*Naja kaouthia*
192		Serum albumin precursor	S59517	3	*Naja naja*
193		Cobra serum albumin	CAA55333	3	*Naja naja*
194	Serum albumin/Alpha-fetoprotein/Afamin	Alpha-fetoprotein, partial	ETE59846	3	*Ophiophagus hannah*
195	Leucine repeat	Leucine-rich repeat neuronal protein 4	XP_007424790	1	*Python bivittatus*
196	Small leucine-rich proteoglycan (SLRP) family	Decorin, partial	ETE60606	1	*Ophiophagus hannah*
197		Leucine-rich repeat and WD repeat-containing protein, partial	ETE61323	1	*Ophiophagus hannah*
198	XPG/RAD2 endonuclease family	Endonuclease domain-containing 1 protein, partial	ETE59939	2	*Ophiophagus hannah*
199		Deoxyribonuclease-2-alpha, partial	ETE73206	1	*Ophiophagus hannah*
200	NHS Family	NHS-like protein 1, partial	ETE71282	1	*Ophiophagus hannah*
201	G-protein coupled receptor	G-protein coupled receptor 161	XP_007428215	1	*Python bivittatus*
202		Putative G-protein coupled receptor	ETE61591	2	*Ophiophagus hannah*
203		Melanocyte-stimulating hormone receptor, partial	ETE69163	1	*Ophiophagus hannah*
204		Latrophilin-2, partial	ETE73569	1	*Ophiophagus hannah*
205		Cadherin EGF LAG seven-pass G-type receptor 2, partial	ETE72621	1	*Ophiophagus hannah*
206		Putative G-protein coupled receptor, partial	ETE70400	1	*Ophiophagus hannah*
207	Zinc finger protein	Thioredoxin domain-containing protein 11, partial	ETE72118	1	*Ophiophagus hannah*
208		Zinc finger protein 91-like isoform X2	XP_007443313	1	*Python bivittatus*
209		Zinc finger protein 687 isoform X1	XP_025027118	1	*Python bivittatus*
210		Zinc finger FYVE domain-containing protein 16, partial	ETE66135	1	*Ophiophagus hannah*
211		Zinc finger and BTB domain-containing protein 14, partial	XP_026555390	1	*Pseudonaja textilis*
212		Zinc finger protein 609 isoform X1	XP_007426825	1	*Python bivittatus*
213	Ras-like protein	Ras GTPase-activating protein 3, partial	ETE71570	1	*Ophiophagus hannah*
214		Rac GTPase-activating protein 1, partial	ETE61861	1	*Ophiophagus hannah*
215		Ras-related protein Rap-2a, partial	ETE66602	1	*Ophiophagus hannah*
216		RalA-binding protein 1, partial	ETE67818	1	*Ophiophagus hannah*
217		Guanylate-binding protein 1-like	XP_007444632	1	*Python bivittatus*
218	Glutathione peroxidase family	Glutathione peroxidase 3, partial	ETE68810	9	*Ophiophagus hannah*
219	Protein family not assigned	Octapeptide-repeat protein T2, partial	ETE65834	1	*Ophiophagus hannah*
220		Atrial natriuretic peptide receptor 2, partial	ETE58463	1	*Ophiophagus hannah*
221		Octapeptide-repeat protein T2, partial	ETE61441	1	*Ophiophagus hannah*
222		GAS2-like protein 2, partial	ETE67730	1	*Ophiophagus hannah*
223		Exocyst complex component 3, partial	ETE60130	1	*Ophiophagus hannah*
224		Vacuolar protein sorting-associated protein 54	ETE70627	1	*Ophiophagus hannah*
225		Cohesin subunit SA-2, partial	ETE63002		*Ophiophagus hannah*
226		Zona pellucida sperm-binding protein 3 receptor, partial	ETE59512	1	*Ophiophagus hannah*
227		Ubiquitin carboxyl-terminal hydrolase 32, partial	ETE63263	1	*Ophiophagus hannah*
228		Putative E3 ubiquitin-protein ligase UBR7	ETE67503	1	*Ophiophagus hannah*
229		Mdm2-binding protein, partial	ETE64533	1	*Ophiophagus hannah*
230		E3 ubiquitin-protein ligase TTC3, partial	ETE73451	1	*Ophiophagus hannah*
231		Protocadherin-23	XP_007425673	1	*Python bivittatus*
232		Nucleolar complex protein 4-like protein, partial	ETE59886	1	*Ophiophagus hannah*
233		Low molecular weight phosphotyrosine protein phosphatase, partial	ETE66708	1	*Ophiophagus hannah*
234		Major histocompatibility complex class I-related protein, partial	ETE56816	1	*Ophiophagus hannah*
235		Beta-2-microglobulin, partial	ETE58426	1	*Ophiophagus hannah*
236		GRAM domain-containing protein 1B, partial	ETE59875	1	*Ophiophagus hannah*
237		von Willebrand factor A domain-containing protein 3B, partial	ETE71898	1	*Ophiophagus hannah*
238		Homeobox protein PKNOX1, partial	XP_007435014	1	*Python bivittatus*
239		Homeobox protein prophet of Pit-1, partial	ETE69018	1	*Ophiophagus hannah*
240		Homeobox protein cut-like 2, partial	ETE71612	1	
241		Inosine-uridine preferring nucleoside hydrolase, partial	ETE68936	1	*Ophiophagus hannah*
242		Signal recognition particle receptor subunit beta	ETE61181	1	*Ophiophagus hannah*
243		Sodium channel protein type 1 subunit alpha	XP_025024892	1	*Python bivittatus*
244		Small serum protein-4	BAJ14709	1	*Gloydius blomhoffii blomhoffii*
245		Clathrin heavy chain 1, partial	ETE68739	1	*Ophiophagus hannah*
246		Neutral amino acid transporter A, partial	ETE71889	1	*Ophiophagus hannah*
247		Bystin	ETE67512	1	*Ophiophagus hannah*
248		Peroxisome biogenesis factor 1-like isoform X1	XP_025032182	1	*Python bivittatus*
249		Dapper-like 1, partial	ETE59781	1	*Ophiophagus hannah*
250		Protein patched-like 2, partial	ETE72035	1	*Ophiophagus hannah*
251		Keratin, type II cytoskeletal 1, partial	ETE67131	1	*Ophiophagus hannah*
252		Keratin, type II cytoskeletal 6A-like	XP_007441333	1	*Python bivittatus*
253		Cytosolic carboxypeptidase 2, partial	ETE72716	1	*Ophiophagus hannah*
254		NADH dehydrogenase subunit 4	YP_003540795	1	*Hypsiglena ochrorhyncha klauberi*
255		Olfactory receptor 2D2-like	XP_007442854	1	*Python bivittatus*
256		Histone-lysine N-methyltransferase SETD1B, partial	ETE63606	1	*Ophiophagus hannah*
257		Helicase SRCAP, partial	ETE66458	1	*Ophiophagus hannah*
258		Tyrosine-protein phosphatase non-receptor type 11-like	XP_015743235	1	*Python bivittatus*
259		Glycerol-3-phosphate acyltransferase 4	ETE64295	1	*Ophiophagus hannah*
260		NEDD4-binding protein 1, partial	ETE71789	1	*Ophiophagus hannah*
261		Nuclear pore complex protein, partial	ETE72717	1	*Ophiophagus hannah*
262		G1/S-specific cyclin-E1, partial	ETE69419	1	*Ophiophagus hannah*
263		Copine-3	ETE62235	1	*Ophiophagus hannah*
264		Disks large-like 1, partial	ETE60775	1	*Ophiophagus hannah*
265		Tumor necrosis factor receptor superfamily member 11B	ETE67452	1	*Ophiophagus hannah*
266		Extracellular matrix protein 1, partial	ETE63009	3	*Ophiophagus hannah*
267		Protein PRRC2C isoform X7	XP_025025988	1	*Python bivittatus*
268		Protein dispatched-like 2, partial	ETE65280	1	*Ophiophagus hannah*
269		Cytoplasmic FMR1-interacting protein 1	ETE70074	1	*Ophiophagus hannah*
270		Sushi domain-containing protein 2 isoform X1	XP_007439094	1	*Python bivittatus*
271		POU domain, class 2, transcription factor 1, partial	ETE68887	1	*Ophiophagus hannah*
272		Vomeronasal type-2 receptor 26-like	XP_015746172	1	*Python bivittatus*
273		snRNA-activating protein complex subunit 4, partial	ETE66257	1	*Ophiophagus hannah*
274		Small subunit processome component 20-like protein, partia	ETE62675	1	*Ophiophagus hannah*
275		Retrotransposon-derived protein PEG10, partial	ETE60414	1	*Ophiophagus hannah*
276		Heterogeneous nuclear ribonucleoprotein R	ETE70095	1	*Ophiophagus hannah*
277		Sacsin, partial	ETE73074	1	*Ophiophagus hannah*
278		Trafficking protein particle complex subunit 3	XP_007439119	1	*Python bivittatus*
279		Putative protein C4orf34	ETE61848	1	*Ophiophagus hannah*
280		Sulfate transporter, partial	ETE72250	1	*Ophiophagus hannah*
281		Solute carrier family 2, facilitated glucose transporter member 11, partial	ETE65979	1	*Ophiophagus hannah*
282		Solute carrier family 25 member 47, partial	ETE64737	1	*Ophiophagus hannah*
283		Citrate synthase, mitochondrial	ETE71902	1	*Ophiophagus hannah*
284		Separin, partial	ETE71706	1	*Ophiophagus hannah*
285		5,6-dihydroxyindole-2-carboxylic acid oxidase, partial	ETE63759	1	*Ophiophagus hannah*
286		Protocadherin-15, partial	ETE73122	1	*Ophiophagus hannah*
287		Tumor necrosis factor receptor superfamily member 11B isoform X2	XP_025019261	1	*Python bivittatus*
288		Microtubule-actin cross-linking factor 1, isoforms 1/2/3/5, partial	ETE72267	1	*Ophiophagus hannah*
289		Ubiquitin carboxyl-terminal hydrolase CYLD	XP_015680147	1	*Protobothrops mucrosquamatus*
290		Peroxidasin, partial	ETE57820	1	*Ophiophagus hannah*
291		Serine palmitoyltransferase small subunit B	XP_025028624	1	*Python bivittatus*
292		C-terminal-binding protein 1, partial	ETE64323	1	*Ophiophagus hannah*
293		StAR-related lipid transfer protein 13	ETE69978	1	*Ophiophagus hannah*
294		Ty3b-i, partial	ETE59080	1	*Ophiophagus hannah*
295		E3 ubiquitin-protein ligase RNF19B, partial	ETE68153	1	*Ophiophagus hannah*
296		PDZ domain-containing protein 6, partial	ETE69093	1	*Ophiophagus hannah*
297		Nebulin, partial	ETE70906	2	*Ophiophagus hannah*
298		Myoferlin, partial	ETE66870	1	*Ophiophagus hannah*
299		Protein mago nashi-like 2	ETE70612	1	*Ophiophagus hannah*
300		H(+)/Cl(-) exchange transporter 7, partial	ETE72134	1	*Ophiophagus hannah*
301		Membrane cofactor protein-like	XP_025021316	2	*Python bivittatus*
302		Holliday junction recognition protein isoform X1	XP_025025001	1	*Python bivittatus*
303		Adenylate cyclase type 2, partial	ETE62750	1	*Ophiophagus hannah*
304		Transmembrane protein, partial	ETE59610	1	*Ophiophagus hannah*
305		Transmembrane protein, partial	ETE58244	1	*Ophiophagus hannah*
306		Type I inositol 3,4-bisphosphate 4-phosphatase	XP_015686159	1	*Protobothrops mucrosquamatus*
307		Complement decay-accelerating factor transmembrane isoform, partial	ETE63384	8	*Ophiophagus hannah*
308		NACHT, LRR and PYD domains-containing protein 6(Belongs to NLRP family)	XP_015679160	1	*Protobothrops mucrosquamatus*
309		Ubiquitin carboxyl-terminal hydrolase 24	ETE67725	1	*Ophiophagus hannah*
310		Epiplakin, partial	ETE58258	1	*Ophiophagus hannah*
311		5’ nucleotidase, partial	AXL95273	1	*Spilotes sulphureus*
312		GTP-binding protein 2, partial	ETE70473	1	*Ophiophagus hannah*
313		Transmembrane protein 41A	XP_007420693	1	*Python bivittatus*
314		Serine/threonine-protein kinase TAO2, partial	ETE67077	1	*Ophiophagus hannah*
315		Serine/threonine-protein kinase WNK1, partial	ETE61641	1	*Ophiophagus hannah*
316		cilia- and flagella-associated protein 57-like, partial	XP_007436852	1	*Python bivittatus*
317		Lymphocyte antigen 6 complex locus protein G6d	ETE61452	1	*Ophiophagus hannah*
318		Histamine H3 receptor, partial	ETE72972	1	*Ophiophagus hannah*
319		Glycerol-3-phosphate acyltransferase 1, mitochondrial, partial	ETE59719	1	*Ophiophagus hannah*
320		Cleft lip and palate transmembrane protein 1-like protein, partial	ETE61569	1	*Ophiophagus hannah*
321		Complement factor B precursor	AAR21601	1	*Naja kaouthia*
322		Selenocysteine lyase	XP_015669194	1	*Protobothrops mucrosquamatus*
323		Serine/threonine-protein kinase Nek1, partial	ETE68306	1	*Ophiophagus hannah*
324		Collagen alpha-1(IV) chain, partial	ETE60834	1	*Ophiophagus hannah*
325		DmX-like protein 2, partial	ETE63888	1	*Ophiophagus hannah*
326		Aldehyde dehydrogenase family 3 member B1, partial	ETE72723	1	*Ophiophagus hannah*
327		Putative ATP-dependent RNA helicase DHX40, partial	ETE68740	1	*Ophiophagus hannah*
328		Immunoglobulin Y2 heavy chain, partial	AFR33766	1	*Python bivittatus*
329		Myomesin-1, partial	ETE65385	1	*Ophiophagus hannah*
330		Cyclic AMP-dependent transcription factor ATF-1, partial	ETE65149	1	*Ophiophagus hannah*
331		Toll-like receptor 4, partial	ETE72495	1	*Ophiophagus hannah*
332		Serine palmitoyltransferase small subunit B	XP_025028624	1	*Python bivittatus*
333		Histone-lysine N-methyltransferase, H3 lysine-79 specific, partial	ETE65559	1	*Ophiophagus hannah*
334		Creatine kinase B-type, partial	ETE69249	1	*Ophiophagus hannah*
335		Fibroblast growth factor 3, partial	ETE69378	1	*Ophiophagus hannah*
336		RB1-inducible coiled-coil protein 1, partial	ETE67067	1	*Ophiophagus hannah*
337		Phosphoinositide 3-kinase regulatory subunit 5, partial	ETE74144	1	*Ophiophagus hannah*
338		Cadherin EGF LAG seven-pass G-type receptor 2, partial	ETE72621	1	*Ophiophagus hannah*
339		Trafficking kinesin-binding protein 1, partial	ETE68220	1	*Ophiophagus hannah*
340		YTH domain family protein 2	ETE65464	1	*Ophiophagus hannah*
341		Vigilin, partial	ETE61946	1	*Ophiophagus hannah*
342		39S ribosomal protein L44, mitochondrial, partial	ETE68399	1	*Ophiophagus hannah*
343		Pseudouridine-5’-monophosphatase, partial	ETE71697	1	*Ophiophagus hannah*
344		Kelch-like protein 13, partial	ETE71947	1	*Ophiophagus hannah*
345		Maleylacetoacetate isomerase	ETE68752	1	*Ophiophagus hannah*
346		Neurexophilin-2, partial	ETE71784	1	*Ophiophagus hannah*
347		Myocyte-specific enhancer factor 2A isoform X1	XP_007425135	1	*Python bivittatus*
348		Membrane cofactor protein-like isoform X1	XP_015743425	1	*Python bivittatus*
349		Ninein-like protein, partial	ETE70166	1	*Ophiophagus hannah*
350		Keratin, type I cytoskeletal 19, partial	ETE70217	1	*Ophiophagus hannah*
351		Intraflagellar transport protein 88-like protein	ETE73657	1	*Ophiophagus hannah*
352		Complement receptor type 2, partial	ETE63383	1	*Ophiophagus hannah*
353		Complement decay-accelerating factor, partial	ETE59511	1	*Ophiophagus hannah*
354		Keratin, type II cytoskeletal 5-like	XP_025030548	1	*Python bivittatus*
355		7-dehydrocholesterol reductase, partial	ETE67784	1	*Ophiophagus hannah*
356		La-related protein 4B	ETE62671	1	*Ophiophagus hannah*
357		Intelectin-1a, partial	ETE57886	1	*Ophiophagus hannah*
358		Cation-independent mannose-6-phosphate receptor	ETE64374	2	*Ophiophagus hannah*
359		Cerebellin-4	ETE65277	1	*Ophiophagus hannah*
360		C3 and PZP-like alpha-2-macroglobulin domain-containing protein 8, partial	ASU45032	1	*Ophiophagus hannah*
361		Neuronal PAS domain-containing protein 2, partial	ETE63668	1	*Ophiophagus hannah*
362		Interferon-induced transmembrane protein 10, partial	ETE66904	1	*Ophiophagus hannah*
363		Myotubularin-related protein 11, partial	ETE72068	1	*Ophiophagus hannah*
364		Tyrosyl-DNA phosphodiesterase 2	XP_026525751	1	*Notechis scutatus*
365		Phosphoinositide 3-kinase regulatory subunit 5, partial	ETE74144	1	*Ophiophagus hannah*

The bold text indicates the proteins identified to have N-terminal acetylation.

**Table 3:** Summary of the venom proteome of *N. oxiana.*

S. No.	Protein Family	Protein	Accession Code	Number of Matched Peptides	Homology with Protein from the Venom of Snake Species	
1	3FTX (Neurotoxin	Neurotoxin homolog NL1	Q9DEQ3	1	*Naja atra*	
2		Short neurotoxin SNTX-1	A6MFK6	1	*Demansia vestigiata*	
3		Neurotoxin II	P01427	1	*Naja oxiana*	
4		Cobrotoxin-b	P80958	1	*Naja atra*	
5		Alpha-cobratoxin	P01391	3	*Naja kaouthia*	
6		Weak toxin 2	Q8AY50	2	*Bungarus candidus*	
7		Weak neurotoxin 6	O42256	1	*Naja sputatrix*	
8		Weak neurotoxin 7	P29181	2	*Naja naja*	
9		Weak toxin S4C11	P01400	1	*Naja melanoleuca*	
**10**		**Muscarinic toxin-like protein 3**	**P82464**	**4**	***Naja kaouthia***	
11		Muscarinic toxin-like protein 2	P82463	4	*Naja kaouthia*	
12		Muscarinic toxin-like protein	Q9W727	1	*Bungarus multicinctus*	
13		Three-finger toxin precursor, partial	ADN67582	1	*Naja atra*	
14		Three-finger toxin precursor, partial	ADN67582	1	*Naja atra*	
15	3FTXs (cytotoxins)	Cytotoxin Vc-5	Q9PS34	2	*Naja oxiana*	
16		Cytotoxin homolog	P14541	1	*Naja kaouthia*	
17		Cytotoxin homolog 5V	Q9W716	1	*Naja atra*	
18		Cytotoxin SP15c	P60308	1	*Naja atra*	
19		Cytotoxin 8	P86540	2	*Naja naja*	
20		Cytotoxin 1	P01447	2	*Naja naja*	
21		Cardiotoxin 7a	Q91126	6	*Naja atra*	
22		Cardiotoxin 1e	AAA90960	2	*Naja atra*	
23	Venom Complement C_3_-like	Venom factor	AAX86641	1	*Austrelaps superbus*	
24		Cobra venom factor	Q91132	10	*Naja kaouthia*	
25		A.superbus venom factor 1	Q0ZZJ6	1	*Austrelaps superbus*	
26		Cobra venom factor alpha chain	Q91132	1	*Naja kaouthia*	
27		Cobra venom factor 1, partial	AXL96620	6	*Ahaetulla prasina*	
28		Cobra venom factor, partial	AWX67646	2	*Boiga irregularis*	
29		Ophiophagus venom factor	I2C090	1	*Ophiophagus hannah*	
30	Venom Kunitz-type family	Kunitz-type serine protease inhibitor	P20229	2	*Naja naja*	
31		BPTI/Kunitz domain-containing protein-like	XP_026546510	1	*Notechis scutatus*	
32		Kunitz/BPTI-like toxin	XP_026579467	1	*Pseudonaja textilis*	
33	natriuretic peptide family	Natriuretic peptide PaNP-c precursor, partial	AAZ82822	1	*Pseudechis australis*	
34	NGF-beta family	Venom nerve growth factor 2	Q5YF89	5	*Naja sputatrix*	
35		Nerve growth factor, partial	AAR24530	1	*Bitis gabonica*	
36		Nerve growth factor	BAN82142	4	*Ovophis okinavensis*	
37		Venom nerve growth factor 2	Q3HXX9	1	*Hoplocephalus stephensii*	
38	ohanin/vespryn family.	Thaicobrin	P82885	1	*Naja kaouthia*	
39		venom PRY-SPRY domain-containing protein, partial	AHZ08803	1	*Micropechis ikaheca*	
40	CRISP	Cysteine-rich venom protein natrin-1	Q7T1K6	3	*Naja atra*	
41		Cysteine-rich secretory protein 1, partial	AXL96607	1	*Ahaetulla prasina*	
42		Cysteine-rich venom protein ophanin	Q7ZT98	1	*Ophiophagus hannah*	
43		Cysteine-rich venom protein, partial	BAP39957	1	*Protobothrops flavoviridis*	
44		Cysteine-rich venom protein 2	Q7ZZN8	1	*Naja atra*	
45	Phosoholipase A2	Acidic phospholipase A2 3	P60045	1	*Naja sagittifera*	
46		Acidic phospholipase A2 2	P00597	1	*Naja kaouthia*	
47		Phospholipase a2	CAA45372	3	*Naja naja*	
48		Neutral phospholipase A2 paradoxin-like beta chain	Q45Z46	2	*Oxyuranus microlepidotus*	
49		Phospholipase A2	AHZ08810	1	*Micropechis ikaheca*	
50		Phospholipase A2	AAA66029.1	1	*Naja naja*	
51		Acidic phospholipase A2 2	P15445	1	*Naja naja*	
52		Acidic phospholipase A2 1	P00596	6	*Naja kaouthia*	
53		Acidic phospholipase A2 1	Q9W7J4	1	*Pseudonaja textilis*	
54		Basic phospholipase A2 T1-2 A chain	P84472	1	*Bungarus candidus*	
55		Acidic phospholipase A2 C	Q92086	11	*Naja sputatrix*	
56		Acidic phospholipase A2 1	P00598	1	*Naja naja*	
57		Acidic phospholipase A2 beta-bungarotoxin A4 chain	P17934	1	*Bungarus multicinctus*	
58		Phospholipase A2 3	P21792	1	*Micrurus nigrocinctus*	
59	Phospholipase B	Phospholipase B, partial	AXL95274	1	*Spilotes sulphureus*	
60		Phospholipase B1, partial	AXL96606	2	*Ahaetulla prasina*	
61		Phospholipase B1, membrane-associated	XP_02653746	1	*Notechis scutatus*	
62	SVMP	Snake venom metalloproteinase	D3TTC2	4	*Naja atra*	
63		Snake venom metalloproteinase	F8RKW1	1	*Drysdalia coronoides*	
64		Snake venom metalloproteinase	Q9PVK7	1	*Naja kaouthia*	
65		Disintegrin and metalloproteinase domain-containing protein 20, partial	ETE72945	1	*Ophiophagus hannah*	
66		Disintegrin and metalloproteinase domain-containing protein 21, partial	ETE71596	1	*Ophiophagus hannah*	
67		disintegrin and metalloproteinase domain-containing protein 10-like, partial	XP_026580760	1	*Pseudonaja textilis*	
68		P-III snake venom metalloprotease, partial	AHZ08819	1	*Micropechis ikaheca*	
69		Zinc metalloproteinase-disintegrin-like kaouthiagin-like	D3TTC1	7	*Naja atra*	
70		Zinc metalloproteinase-disintegrin-like atrase-A	D5LMJ3	14	*Naja atra*	
71		Hemorrhagic metalloproteinase-disintegrin-like kaouthiagin	P82942	2	*Naja kaouthia*	
72		metalloproteinase 7, partial	AXL96626	1	*Ahaetulla prasina*	
73		metalloproteinase, partial	AWX67576	1	*Boiga irregularis*	
74		Snake venom metalloproteinase-disintegrin-like mocarhagin	Q10749	3	*Naja mossambica*	
75		Snake venom metalloproteinase	Q9W6M5	1	*Deinagkistrodon acutus*	
76	Snake venom serine proteinase (peptidase S1 family)	Tissue-type plasminogen activator, partial	ETE66683	3	*Ophiophagus hannah*	
77		tissue-type plasminogen activator, partial	XP_026544671	2	*Notechis scutatus*	
78		Snake venom serine protease 3	O13058	1	*Protobothrops flavoviridis*	
79		Serine protease 27, partial	ETE64653	1	*Ophiophagus hannah*	
80		Thrombin-like enzyme TLP	P86545	1	*Naja naja*	
81		Snake venom serine protease 3	AAG10790	1	*Protobothrops jerdonii*	
82		Snake venom serine protease Dav-PA	Q9I8X1	1	*Deinagkistrodon acutus*	
83		serine protease 53	XP_026576912	1	*Pseudonaja textilis*	
84	5’-nucleotidase family	5’ nucleotidase, partial	AXL95273	1	*Spilotes sulphureus*	
85	Aminopeptidase	aminopeptidase N isoform X2	XP_026565037	4	*Pseudonaja textilis*	
86	type-B carboxylesterase/lipase	acetylcholinesterase	XP_026549820	1	*Notechis scutatus*	
87	Phosphodiesterase	Phosphodiesterase	BAN89425	2	*Ovophis okinavensis*	
88		Phosphodiesterase partial	ALA20853	1	*Macropisthodon rudis*	
89		Phosphodiesterase partial	AXL96599	1	*Borikenophis portoricensis*	
90		Ectonucleotide pyrophosphatase/phosphodiesterase family member 3 isoform X2	XP_026561286	2	*Pseudonaja textilis*	
91		Snake venom Phosphodiesterase	A0A2D0TC04	2	*Naja atra*	
92	Flavin monoamine oxidase family	L-amino acid oxidase, partial	AVX27607	7		*Naja atra*
93		L-amino-acid oxidase	XP_026538830	4		*Notechis scutatus*
94		L-amino-acid oxidase	Q4JHE3	1		*Oxyuranus scutellatus scutellatus*
95		L-amino-acid oxidase	Q4JHE1	1		*Pseudechis australis*
96		L-amino-acid oxidase	A8QL58	1		*Naja atra*
97	True venom lectin family	C-type lectin Cal	P21963	1		*Crotalus atrox*
98	Glutathione peroxidase	Glutathione peroxidase 3, partial	ETE68810	1		*Ophiophagus hannah*
99		Glutathione peroxidase 3 isoform X1	XP_026541908	1		*Notechis scutatus*
100		Glutathione peroxidase 3 isoform X1	XP_026552406	1		*Pseudonaja textilis*
101	Leucine repeat	Leucine-rich repeat and death domain-containing protein 1	XP_026543987	1		*Notechis scutatus*
102	TNF receptor superfamily	Tumor necrosis factor receptor superfamily member 11B	XP_026545353	1		*Notechis scutatus*
103		Tumor necrosis factor receptor superfamily member 11B	XP_026559377	1		*Pseudonaja textilis*
104		Tumor necrosis factor receptor superfamily member 11B	ETE67452	1		*Ophiophagus hannah*
105	Intermediate filament family	Keratin, type II cytoskeletal 1, partia	ETE67131	1		*Ophiophagus hannah*
106		Keratin, type II cytoskeletal 4-like	XP_026539658	1		*Notechis scutatus*
107		Keratin, type II cytoskeletal 5, partial	ETE59039	1		*Ophiophagus hannah*
108		Keratin, type II cytoskeletal 5, partial	ETE59038	2		*Ophiophagus hannah*
109		Keratin, type II cytoskeletal 1-like	XP_026573193	1		*Pseudonaja textilis*
110		Keratin, type I cytoskeletal 19, partial	ETE70217	2		*Ophiophagus hannah*
111		Keratin, type I cytoskeletal 18-like isoform X1	XP_026521302	1		*Notechis scutatus*
112	Serpin Family	Serpin B5, partial	ETE65002	1		*Ophiophagus hannah*
113	Ankyrin repeat domain	M-phase phosphoprotein 8, partial	ETE73652	1		*Ophiophagus hannah*
114	Zinc finger containing proteins	Zinc finger protein, partial	ETE62318	1		*Ophiophagus hannah*
115		Zinc finger protein, partial	ETE62303	1		*Ophiophagus hannah*
116		Zinc finger protein 804A	XP_026552505	1		*Pseudonaja textilis*
117		Zinc finger SWIM domain-containing protein 6	XP_026572863	1		*Pseudonaja textilis*
118		Zinc finger MYM-type protein 2 isoform X1	XP_026564670	1		*Pseudonaja textilis*
119		Zinc finger BED domain-containing protein 1	XP_026522663	1		*Notechis scutatus*
120	NHS Family	NHS-like protein 1 isoform X1	XP_026561348	1		*Pseudonaja textilis*
121	Protein family not assigned	Holliday junction recognition protein	XP_026519764	1		*Notechis scutatus*
122		N-acetylgalactosaminyltransferase 7 isoform X1	XP_026555474	1		*Pseudonaja textilis*
123		PHD finger protein 3	XP_026520899	1		*Notechis scutatus*
124		Sulfhydryl oxidase 1(contains FAD binding domain)	ETE70041	1		*Ophiophagus hannah*
125		C-C chemokine receptor type 10, partial	ETE65216	1		*Ophiophagus hannah*
126		Cytosolic carboxypeptidase 2	XP_026521145	1		*Notechis scutatus*
127		SUMO-specific isopeptidase USPL1 isoform X1	XP_026564646	1		*Pseudonaja textilis*
128		Protein VPRBP	ETE70381	1		*Ophiophagus hannah*
129		Cilia- and flagella-associated protein 97	XP_026553667	1		*Pseudonaja textilis*
130		lpxK, partial	ETE68446	1		*Ophiophagus hannah*
131		Zinc phosphodiesterase ELAC protein 2, partial	ETE70777	1		*Ophiophagus hannah*
132		NHS-like protein 1 isoform X1	XP_026561348	1		*Pseudonaja textilis*
133		Pro-cathepsin H	XP_026565144	1		*Pseudonaja textilis*
134		C4b-binding protein alpha chain-like isoform X1	XP_026571379	2		*Pseudonaja textilis*
135		Janus kinase and microtubule-interacting protein 3 isoform X1	XP_026566312	1		*Pseudonaja textilis*
136		WD and tetratricopeptide repeats protein 1	XP_026558310	1		*Pseudonaja textilis*
137		Pro-cathepsin H	XP_026565144	1		*Pseudonaja textilis*
138		C4b-binding protein alpha chain-like isoform X1	XP_026571379	2		*Pseudonaja textilis*
139		Janus kinase and microtubule-interacting protein 3 isoform X1	XP_026566312	1		*Pseudonaja textilis*
140		WD and tetratricopeptide repeats protein 1	XP_026558310	1		*Pseudonaja textilis*

The bold text indicates the proteins identified to have N-terminal acetylation.

## Supplementary Materials

The following are available online at https://www.mdpi.com/2072-6651/12/11/669/s1 Table S1: De novo peptide sequencing Naja naja venom. Table S2: De novo peptide sequencing Naja oxiana venom. Table S3: Proteomic data of Naja naja venom searched against Serpents Uniprot protein data base. Table S4: Proteomic data of Naja naja venom searched against King cobra Uniprot protein data base. Table S5: Proteomic data of Naja oxiana venom searched against Serpents Uniprot protein data base. Table S6: Proteomic data of *Naja oxiana* venom searched against Serpents Uniprot protein data base.
